# *Labrenzia* sp. BM1: A Quorum Quenching Bacterium That Degrades *N*-acyl Homoserine Lactones via Lactonase Activity

**DOI:** 10.3390/s140711760

**Published:** 2014-07-03

**Authors:** Norshazliza Ab Ghani, Siti Nur Maisarah Norizan, Xin Yue Chan, Wai-Fong Yin, Kok-Gan Chan

**Affiliations:** Division of Genetics and Molecular Biology, Institute of Biological Sciences, Faculty of Science, University of Malaya, Kuala Lumpur 50603, Malaysia; E-Mails: norshazliza_shaz@ymail.com (N.A.G.); outfeet@yahoo.com (S.N.M.N.); xinyuechan@gmail.com (X.Y.C.); yinwaifong@yahoo.com (W.-F.Y.)

**Keywords:** *Labrenzia* sp., quorum quenching, quorum sensing, marine, seawater, rapid resolution liquid chromatography (RRLC), *N*-acylhomoserine lactone, *N*-(3-oxohexanoyl)-l-homoserine lactone

## Abstract

We report the degradation of quorum sensing *N*-acylhomoserine lactone molecules by a bacterium isolated from a Malaysian marine water sample. MALDI-TOF and phylogenetic analysis indicated this isolate BM1 clustered closely to *Labrenzia* sp. The quorum quenching activity of this isolate was confirmed by using a series of bioassays and rapid resolution liquid chromatography analysis. *Labrenzia* sp. degraded a wide range of *N*-acylhomoserine lactones namely *N*-(3-hexanoyl)-l-homoserine lactone (C6-HSL), *N*-(3-oxohexanoyl)-l-homoserine lactone (3-oxo-C6-HSL) and *N*-(3-hydroxyhexanoyl)-l-homoserine lactone (3-hydroxy-C6-HSL). Re-lactonisation bioassays confirmed *Labrenzia* sp. BM1 degraded these signalling molecules efficiently via lactonase activity. To the best of our knowledge, this is the first documentation of a *Labrenzia* sp. capable of degrading *N*-acylhomoserine lactones and confirmation of its lactonase-based mechanism of action.

## 1. Introduction

Quorum sensing (QS) is a form of cell-cell communication system that allows bacteria to communicate via the production and reception of small diffusible signal molecules, thereby regulating gene expression in response to bacterial cell density [1,2]. Once sufficient concentration of the signal has been reached, gene expression will be modulated either directly by interacting with a transcriptional regulator or indirectly by activating a signaling cascade [3,4]. QS was first discovered as a mechanism that regulated bioluminescence in *Vibrio fischeri.* Since then, QS has been documented in many different bacteria and various types of signal molecules have been identified [5–7].

Three types of QS molecules exist in bacteria, which are (1) LuxI/LuxR-type QS in proteobacteria which use *N*-acylhomoserine lactones (AHL) as signaling molecules; (2) the *luxS*-encoded autoinducer-2 (AI-2) system that exists in both Gram-positive and Gram-negative bacteria; (3) post-translationally modified oligopeptide-two-component-type QS in most Gram-positive bacteria [5]. However, the types that are most widely investigated are AHL and AI-2 [5,8]. QS control several phenotypes such as bioluminescence, biofilm, swarming, which has been shown to contribute to bacterial pathogenesis [9–11]. Therefore, it is not surprising that the study of QS systems has important implications in the control of microbial infections.

On the other hand, quorum quenching (QQ) refers to the disruption of the bacterial QS process. It was first reported by Dong *et al.* in a Gram-positive *Bacillus* species which inactivates AHLs via lactonase activity [12]. Since then, QQ mechanisms have been found in various organisms to countermeasure the benefit that QS brings to their competitors [13]. QQ can be achieved through enzymatic inactivation of AHL either by detachment of the *N*-acyl side chain from the lactone ring via acylase or the opening of the lactone ring moiety by AHL lactonase, and both ways will inactivate the AHLs [13–16].

In a polymicrobial environment such as that of the marine ecosystem, QS bacteria are likely to gain competitive advantage as they can react to stimuli and compete in population-density synchronized manner. Therefore, to overcome this, competitors to QS bacteria that are equipped with QQ ability will be able to countermeasure the benefits accorded by the QS mechanism. Moreover, QS controls several phenotypes, especially virulence factors, and QQ has been regarded as a promising novel anti-infective therapy [13,14]. In view of this, we therefore investigated the presence of QQ bacteria in Malaysian marine water samples with the hope of isolating novel QQ bacteria and exploring their QQ enzymes for downstream application such as attenuation of QS phenotypes [13].

## 2. Experimental Section

### 2.1. Collection of the Malaysian Seawater Sample

One seawater sample (50 mL) was collected using sterile polypropylene tubes from the shoreline of Batu Maung, Penang (N 05°18.6791′, E 100°17.971′), Malaysia. The pH of the collected marine water sample was recorded as pH 8. The seawater sample was immediately processed upon returning to the laboratory. To process the sample, the water sample was serially diluted and spread onto LB agar. Bacteria with observable different morphology were isolated after incubation for 24 h at 28 °C. Pure colonies were obtained with several passages on LB agar.

### 2.2. Bacterial Strains and Culture Conditions

*Bacillus cereus* and *Escherichia coli* TOP10 cells were used as positive and negative controls, respectively, for AHL degradation bioassays [17]. *Chromobacterium violaceum* CV026, an AHL biosensor, was also used in this study to detect the presence of short chain AHLs by the formation of a purple pigment when short chain AHLs are detected [18]. *E. coli* TOP10 cell was used as host for cloning. All bacteria were cultured at 28 °C in LB media, except for *E. coli* TOP10 which was cultured at 37 °C in LB media.

### 2.3. MALDI-TOF-Mass Spectrometry (MALDI-TOF-MS) Identification of Bacteria

Bacterial isolates were identified using a MALDI-TOF-MS (Bruker, Leipzig, Germany) extraction method with UV laser wavelength of 337 nm and acceleration voltage of 20 kV [19–21]. The spectra were then analyzed in the Bruker MALDI Biotyper Real Time Classification (RTC) Version 3.1 (Build 65) software. The dendrogram was generated using the standard MALDI Biotyper MSP creation method [19].

### 2.4. Phylogenetic Analysis of 16S rRNA Genes Sequences

Genomic DNA of isolate BM1 was extracted using QIAamp^®^ DNA Mini Kit (QIAGEN GmBH, Hilden, Germany) and used for PCR as DNA template. 16S rRNA genes were PCR-amplified with the forward primer 27F (5′-AGAGTTTGATCMTGGCTCAG-3′), and the reverse primer 1525R (5′AAGGAGGTGWTCCARCC-3′), as described previously [22]. Briefly, The PCR cycles consisted of an initial denaturation at 94 °C for 5 min, followed by 30 cycles at 94 °C for 30 s, annealing at 63 °C for 30 s and extension at 72 °C for 1 min 30 s, and a final extension at 72 °C for 5 min. Gene sequences were compared with GenBank databases using the BLASTN program followed by sequence alignment and phylogenetic analyses using the Molecular Evolutionary Genetic Analysis (MEGA) version 5.1 with parameter Neighbour-Joining algorithm and bootstrap 1000 re-samplings [23]. Identification of bacterial isolate and their accession numbers acquired from GenBank.

### 2.5. AHL Inactivation Assay

An AHL inactivation assay was conducted to screen bacterial isolates capable of degrading AHLs [15,24]. Bacterial cells were harvested by centrifugation, cell pellets were washed twice and resuspended in PBS buffer (100 mM, pH 6.5). Selected synthetic AHLs (C6-HSL, 3-oxo-C6-HSL and 3-hydroxy-C6-HSL, Sigma-Aldrich, St. Louis, MO, USA) of various concentrations were dispensed into sterile micro-centrifuge tubes and dried by evaporation. Cells suspension (in PBS buffer) was added to rehydrate the AHL to the final concentration of 0.5 μM. Mixtures were then incubated at 28 °C with shaking at 220 rpm for 0 h and 24 h. Aliquots of AHLs were withdrawn at 0 h and 24 h and residual AHLs were detected using *C. violaceum* CV026. AHL aliquots were withdrawn at 0 h, 24 h (with and without HCl-treatment) after incubation with the QQ cells were analysed by using rapid resolution liquid chromatography (RRLC) and assayed with biosensor. For AHL inactivation assay, reaction mixtures were stopped by heat inactivation at 95 °C for 5 min and 10 μL of the reaction mixture was spotted onto sterile paper disc placed on *C. violaceum* CV026 lawn and incubated overnight at 28 °C. Reduction or abolishment of purple pigments after 24 h of incubation indicates AHL degradation. PBS and *B. cereus* served as negative and positive controls, respectively. To confirm whether *Labrenzia* sp. BM1 degraded AHLs via lactonase activity, we acidified the AHL degradation mixture to promote re-lactonisation of the opened lactone rings [25]. Formation of purple color pigmentation after the addition of 0.2 M HCl indicated lactonase production [15].

### 2.6. Rapid Resolution Liquid Chromatography (RRLC) Analysis

Sample preparation for RRLC analysis was performed similar to the whole-cell AHLs inactivation assay described. AHLs were extracted twice using ethyl acetate, followed by drying in the fume hood before resuspension in 100 μL of acetonitrile for RRLC analysis. AHLs degradation was analyzed using an Agilent Technologies (Agilent 1200 series, Waldbronn, Germany) 1200 series RRLC system, as described previously [26]. Briefly, AHLs samples were separated using an Agilent Poroshell120 EC-C18 column (4.6 mm × 100 mm, packed with 2.7 μm particle size) with the elution procedure consisting of an isocratic profile of acetonitrile/water (35:65, v/v). Flow rate of 0.7 mL/min was applied and AHL detection was carried out at 210 nm [6]. Known amounts of synthetic AHLs (C6-HSL), (3-oxo-C6-HSL) and (3-hydroxy-C6-HSL) were included as standards. AHL incubated with *E. coli* TOP10 cells and PBS served as a negative controls. Experiments were performed in duplicate.

## 3. Results and Discussion

### 3.1. Isolation and Identification of Strains

In this study, we have isolated four different bacteria colonies from our Malaysian shoreline marine water samples. MALDI-TOF-MS and 16S rRNA was performed to identify the isolates. Of these samples, BM1 showed high degree of degradation of various *N*-acylhomoserine lactones and hence was chosen for further work.

**Figure 1.**
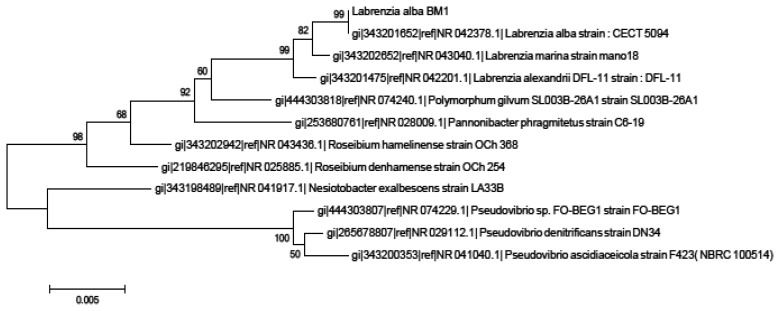
Phylogenetics analysis of isolate BM1. Tree was generated using MEGA 5.1. Isolate BM1 clustered closely to *Labrenzia* sp.

Isolate BM1 could not be identified due to the lack of reference spectra in the database that limits the identification of this sample to the genus or species level [27]. Hence, we proceeded to the molecular identification using phylogenetic trees generated on BM1 16S rRNA gene nucleotide sequences (accession number KJ 880043) which showed that our isolate BM1 is closely related to *Labrenzia* sp. with high bootstrap value (99%) (Figure 1).

### 3.2. Degradation of AHLs and the Mechanism of QQ by Labrenzia sp. BM1

**Figure 2.**
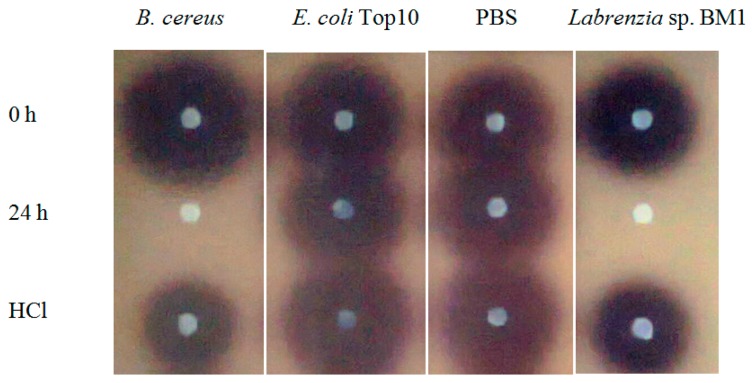
Degradation of C6-HSL by *Labrenzia* sp. BM1. C6-HSL was incubated with bacterial cell suspensions for 0 h, 24 h and HCl was added into aliquot incubated with QQ cells for 24 h. *B. cereus* served as positive control whereas *E. coli* Top10 and PBS were used as negative controls. The purple pigments after addition of HCl suggested re-lactonisation of the digested AHL, and this indicated that *Labrenzia* sp. BM1 produced lactonase which inactivated the tested AHLs.

*Labrenzia* sp. BM1 showed significant degradation of C6-HSL which was depicted by the reduction or disappearance of the purple pigments after 24 h of incubation (Figure 2). Furthermore, *Labrenzia* sp. BM1 cells were also tested for their ability to degrade other types of AHLs including *N*-(3-oxo-hexanoyl)-l-homoserine lactone (3-oxo-C6-HSL) and *N*-(3-hydroxyhexanoyl)-l-homoserine lactone (3-hydroxy-C6-HSL), which represent AHLs with oxo and hydroxy groups, respectively (Table 1). Our data suggested that *Labrenzia* sp. BM1 could efficiently degrade both 3-oxo-C6-HSL and 3-hydroxy-C6-HSL.

**Figure 3.**
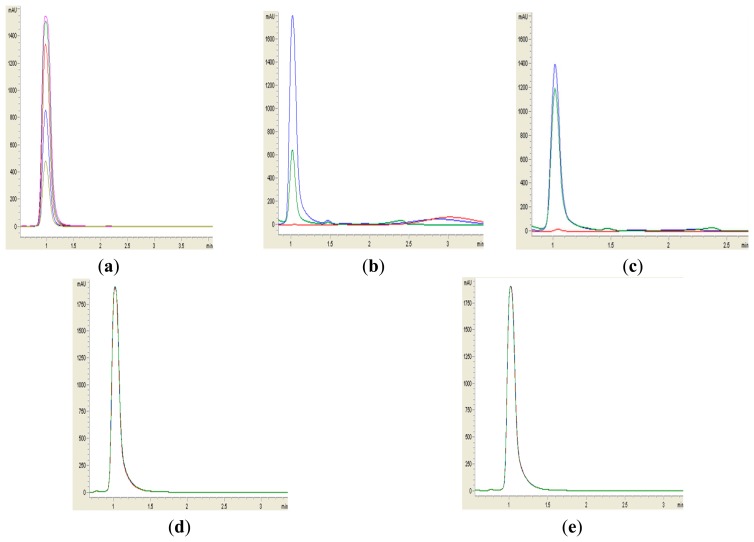
RRLC analysis of 3-oxo-C6-HSL degradation. Residual 3-oxo-C6-HSL (with elution time of 1.00 ± 1.2 s), after degradation for 0 h (blue), 24 h (red) and relactonisation with HCl (green), was monitored at OD 210 nm. Degradation of 3-oxo-C6-HSL was depicted by the reduction of milliabsorbance unit (mAU) in the chromatogram. Samples containing standards 3-oxo-C6-HSL (0.2 μg/μL, 0.4μg/μL, 0.6 μg/μL, 0.8 μg/μL and 1.0 μg/μL) (corresponding to peaks with ascending height) (**a**) sample BM1 (**b**), *B. cereus* acted as positive control (**c**), *E. coli* Top10 (**d**), PBS buffer acted as the negative controls (**e**). Results show significant degradation and relactonisation of 3-oxo-C6-HSL by *Labrenzia* sp. BM1.

Formation of purple pigmentation after the addition of 0.2 M HCl indicated *Labrenzia* sp. BM1 inactivated AHL molecules via the enzyme lactonase [28]. When the opened ring AHL was acidified, this will promote re-lactonisation of the opened ring into intact AHLs [28]. RRLC was then conducted to further confirm the AHLs degradation activity via a lactonase mechanism (Figures 2 and 3). Using a similar approach, we used RRLC to analyse degradation of other AHLs and the data are summarised in Table 2. Figure 3 shows the representative chromatograms. Based on the whole-cell AHL inactivation assay and RRLC analyses on degradation of various AHL as lactonase substrate, strong QQ activity was observed in *Labrenzia* sp. BM1 (Figure 3, Table 2).

Many QQ bacteria have been isolated from tropical marine environments [26]. For example, we have previously described a marine pseudomonad that shows both QS and QQ activities. In this work, we extended the search of marine QQ bacteria in another sampling spot with the aim to identify its QS interference ability and its QQ mechanism. Here, we described the isolation of *Labrenzia* sp. BM1 and its QQ properties were confirmed.

To date, many QQ genes have been well documented, for example AiiA lactonase homologs of *Bacillus* and PvdQ and QuiP of *Psudomonas* have been well characterized [29]. Various lactonase activities have been reported in many proteobacteria such as AiiA, AttM, AiiB [12,30] and AhlD [31]. There is also another class of AHL-degrading enzymes which is amidase/acylase exemplified by AiiD [13,29] that degrade AHLs by detaching the *N*-acyl side chain from the homoserine lactone. Under alkaline conditions AHLs are rapidly inactivated by pH-dependent lactonolysis in which the *N*-acyl- homoserine lactone ring is hydrolysed to the ring-opened form, a reaction that be reversed by acidification [8,9]. On the other hand, *Acinetobacter* sp. has shown a broad activity in degrading short chain AHLs via lactonase [15]. Interestingly, this is the first report that shows *Labrenzia* sp. BM1 exhibiting QQ activity. Our work presented here has added a new member of the *Labrenzia* sp. to the expanding list of QQ bacteria. This finding provides a basis to search for more QQ marine bacteria as most current work is focusing on QS mechanisms in marine bacteria. We hypothesise that QQ bacteria are important in the marine environment as they will be responsible for the turnover of AHLs thus preventing overaccumulation of AHLs in marine microbial niches such as biofilms.

*Labrenzia* sp. (formally classified as *Stappia* a*lba*) has been studied for its general biochemical properties [32] but its QQ mechanism has not been documented before. Our data show for the first time, that *Labrenzia* sp. BM1 efficiently degraded AHLs. Since most Gram-negative bacteria use QS to modulate many phenotypes, including virulence determinants such biofilm formation, our work may path the way to explore *Labrenzia* sp. BM1 for biocontrol in aquaculture by destruction of QS signal molecules in marine QS pathogens and to attenuate them without the use of antibiotics [13,14], or prevention of biofouling. *Labrenzia* sp. BM1 may also serve as a model microorganism for microbial ecology studies of QS and QQ systems in the marine habitat.

## 4. Conclusions

We report here the QQ activity of *Labrenzia* sp. BM1 isolated from a Malaysian marine water sample. Our data demonstrated that *Labrenzia* sp. BM1 significantly degraded various *N*-acylhomoserine lactones (C6-HSL, 3-oxo-C6-HSL and 3-hydroxy-C6-HSL). Further investigations are being carried out to clone the QQ gene and to study the ecological role of QQ gene *Labrenzia* sp. BM1 in its marine environment.

## Figures and Tables

**Table 1.** Summary of AHLs inactivation assays detected using CV026 overlay.

**AHLs**	***B. cereus* (Positive Control)**	***E. coli* Top10 (Negative Control)**	**PBS Buffer (Negative Control)**	***Labrenzia* sp. BM1**
C6-HSL	+	−	−	+
3-oxo-C6-HSL	+	−	−	+
3-hydroxy-C6-HSL	+	−	−	+

+ denotes AHL was degraded, − denotes no AHL degradation was observed.

**Table 2.** Summary of AHLs degradation analysed using Rapid Resolution Liquid Chromatography (RRLC).

**Time/Samples**	**AHLs**	**0 h (mAU)**	**24 h (mAU)**	**HCl (mAU)**
*Labrenzia* sp. BM1	3-oxo-C6-HSL	1700	0	650
*B. cereus* (positive control)		1400	0	1200
*E. coli* Top10 (negative control)		1900	1900	1900
PBS buffer (negative control)		1900	1900	1900

*Labrenzia* sp. BM1	3-hydroxy-C6-HSL	200	0	85
*B. cereus* (positive control)		200	0	145
*E. coli* Top10 (negative control)		200	200	200
PBS buffer (negative control)		200	200	200

*Labrenzia* sp. BM1	C6-HSL	145	20	65
*B. cereus* (positive control)		165	0	40
*E. coli* Top10 (negative control)		170	170	170
PBS buffer (negative control)		200	200	200

AHLs degradation was depicted as reduction of milli-absorbance unit (mAU).
